# Mapping the genomic landscape of *Prunus* spp. with PrunusMap

**DOI:** 10.1093/hr/uhae301

**Published:** 2024-10-24

**Authors:** Najla Ksouri, María Ángeles Moreno, Bruno Contreras-Moreira, Yolanda Gogorcena

**Affiliations:** Group of Genomics of Fruit Trees and Grapevine, Department of Pomology, Estación Experimental de Aula Dei-Consejo Superior de Investigaciones Científicas, Avenida de Montañana 1005, E50059 Zaragoza, Spain; Group of Fruit Tree Breeding and Fuit Quality, Department of Pomology, Estación Experimental de Aula Dei-Consejo Superior de Investigaciones Científicas, Avenida de Montañana 1005, E50059 Zaragoza, Spain; Laboratory of Computational and Structural Biology, Department of Genetics and Plant Breeding, Estación Experimental de Aula Dei-Consejo Superior de Investigaciones Científicas, Avenida de Montañana 1005, E50059 Zaragoza, Spain; Group of Genomics of Fruit Trees and Grapevine, Department of Pomology, Estación Experimental de Aula Dei-Consejo Superior de Investigaciones Científicas, Avenida de Montañana 1005, E50059 Zaragoza, Spain

## Abstract

Next-generation sequencing has fueled significant advancement in plant breeding tools, such as genome-wide association studies and single-nucleotide polymorphism (SNP) analysis. In this dynamic landscape, plant databases housing SNP markers have evolved into hubs facilitating breeding initiatives and genomic research. PrunusMap, accessible at https://prunusmap.eead.csic.es is an open-source Web application tailored for the *Prunus* community. Featuring a user-friendly interface, PrunusMap empowers users to seamlessly align and locate markers across multiple genome versions of *Prunus* species and cultivars, supporting different queries and formats. Beyond locating marker positions, it provides a comprehensive list of annotated nearby genes and proteins. This streamlined process, driven by four intuitive features ‘Find markers’, ‘Align sequences’, ‘Align proteins’, and ‘Locate by position’, significantly reduces workload and boosts efficiency, particularly for users with limited bioinformatics expertise. Moreover, PrunusMap’s versatility is underscored by its commitment to incorporate additional *Prunus* genome sequences, annotations, and markers upon user request.

## Introduction

In the Rosaceae family, the availability of 74 whole genomes and annotations provide a robust foundation for marker development (https://www.plabipd.de/plant_genomes_pa.ep, last accessed July 2024). Restriction fragment length polymorphism (RFLP) markers were instrumental in producing a saturated map for almond, the first and most comprehensive in the stone fruit genus *Prunus.* Subsequently, randomly amplified polymorphic DNA (RAPD) markers found widespread use in germplasm diversity studies in peach and other *Prunus* species, helping map loci controlling traits such as flesh color and fruit texture [1]. The development of amplified RFLP (AFLP) markers revealed associations with traits such as resistance root-knot nematodes [2] and evergreen [3]. However, the low reproducibility (RAPDs) and high cost (RFLPs, AFLPs) of these markers led to their replacement by SSRs and SNPs. While SSR markers find frequent utility in *Prunus* breeding, SNP markers have gained prominence due to their cost-effectiveness, high throughput, and genome-wide coverage [4, 5]. Moreover, the accurate prediction of marker positions and the identification of nearby genes are critical for understanding the genetic mechanisms underlying target traits and accelerating modern breeding cycles. For instance, research on Sharka disease in peach identified three highly significant associated SNPs on chromosomes 2 and 3, conferring a reduction in susceptibility to Plum pox virus. The *Prupe.2G065600* gene on chromosome 2, encoding an RTM2-like protein was selected as a major effect candidate gene [6]. The emergence of SNP markers has indeed revolutionized *Prunus* breeding with two main repositories cataloging the genetic variants: the PeachVar-DB portal [7] and the Genome Database for Rosaceae (GDR) [8–10]. The PeachVar-DB portal provides different tools to retrieve SNPs and Indels extracted from whole genome sequence libraries of peach and wild relatives [7]. Users can conveniently access these variants by specifying either a specific gene identifier or a genomic region of interest, with all coordinates extracted from the peach reference genome version 2.0. In contrast, the GDR stands out as a more multifaceted database, encompassing a broad spectrum of genomic and genetic data within the Rosaceae family. It provides a diverse range of tools aimed at exploring these resources. However, it lacks in capturing high-density genomic data obtained from resequencing projects [7]. Moreover, browsing the GDR often requires switching between multiple pages, which can be cumbersome.

A friendly tool to map the location of genetic markers rapidly and accurately, along with information about nearby genes, could greatly assist breeders, particularly those with limited bioinformatics expertise. To address this need, we developed PrunusMap. Its objective is to streamline the process of locating *Prunus* markers on both genetic and physical maps, accommodating various input formats. Currently, PrunusMap curates SNPs for a variety of *Prunus* species, including relevant peach cultivars (e.g. ChineseCling, Zhongyoutao14, 124Pan, and Sovetskiy) and wild relatives such as *Prunus davidiana*, *P. ferganensis*, *P. kansuensis*, and *P. mira*. Additionally, it supports other economically valuable species like almond (*P. dulcis*), sweet cherry (*P. avium*), apricot (*P. armeniaca*), and Japanese apricot (*P. mume*).

PrunusMap is accessible at https://prunusmap.eead.csic.es and offers four key features for data retrieval:

1. ‘Find markers’: to retrieve the position of markers by providing their identifiers.
2. ‘Align sequences’: to obtain the position of FASTA sequences (FAST-All, It is a format for sequences) by pairwise alignment.
3. ‘Align proteins’: to determine the position of FASTA amino acid sequences considering splicing and frameshift variations.
4. ‘Locate by position’: to examine specific loci based on their map position, to identify which genes, markers, or other loci are present in those regions.

**Figure 1:**
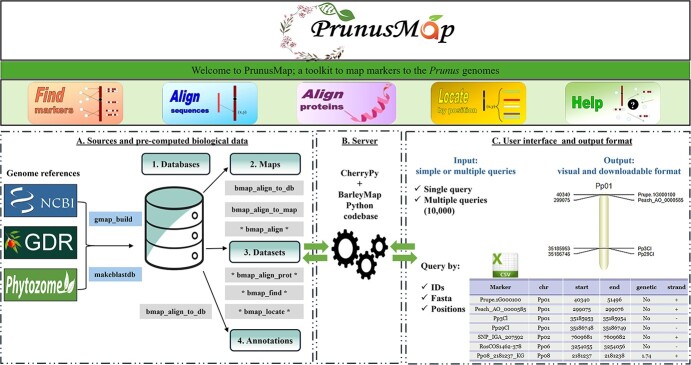
Workflow of PrunusMap Web application. The reference genome sequences of *Prunus* species and cultivars were downloaded from NCBI, GDR, and Phytozome. Gmap_build and makeblastdb command line executables were used to build the corresponding databases from the FASTA files. Databases, maps, datasets, and annotations represent the essential types of biological data required for PrunusMap configuration. Gray-highlighted boxes correspond to tools that are activated once each of the biological resources is properly set up (exp: “bmap_align_to_db” tool is activated once the database is well configured). Those tagged with asterisks correspond to web-accessible tools, while the rest correspond to the standalone commands. PrunusMap accepts both simple or multiple queries as input, and the results are displayed in visual and downloadable formats (CSV: Comma-Separated Values).

PrunusMap is a fork of Barleymap, a tool originally designed to serve the barley community [11], with two key enhancements: (i) it accommodates a range of *Prunus* species and cultivars, catering to the diverse needs of *Prunus* research community, and (ii) it introduces the novel functionality of mapping protein sequences, which are usually more conserved than nucleotide sequences. The data flow and architecture are summarized in Fig. 1.

## Results

### Comparing GMAP and BLASTN

**Figure 2:**
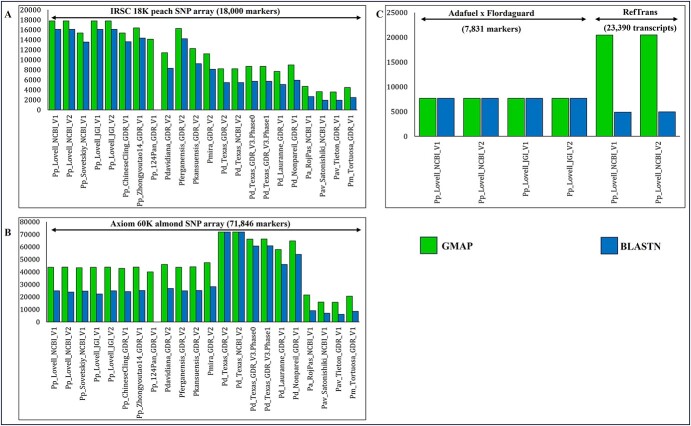
Performance comparison of GMAP and BLASTN aligners. (A) and (B) correspond to the alignment of the IRSC 18 K peach and 60 K almond arrays against all PrunusMap databases, respectively. (C) corresponds to the alignment of genetic markers and transcripts. The *x*-axis refers to the different databases (maps according to PrunusMap terminology) used for the alignment, while the *y*-axis corresponds to the number of sequences.

The performance of Genomic Mapping and Alignment Program (GMAP) and Nucleotide BLAST (BLASTN) was assessed by aligning marker sequences against various *Prunus* databases. As shown in Fig. 2 and Fig. S1, GMAP consistently outperformed BLASTN in terms of mapped maker counts, regardless of the SNP array or the reference database used. As expected, the alignment rate was consistently higher for maps targeting the same species as the array. For instance, the peach 9 K and 18 K arrays exhibited a greater number of successfully aligned SNPs against peach databases compared to those of more distantly related species. Notably, the alignment rate was even higher when these arrays were aligned against the *P. persica* cv. Lovell, which was used as the reference genome to develop these arrays (Figs 2A and S1A). Similarly, SNP markers from the 60 K almond array mostly matched the Texas_v2 databases compared to Texas v3, Lauranne, and Nonpareil cultivars (Fig. 2B**)**. This is likely due to the array design, which involved the alignment of resequenced raw reads against Texas_v2 prior to SNP calling [12].

The 6 K cherry SNP array exhibited a bias toward the peach genome (cv. Lovell) (Fig. S1B). This discrepancy is likely due to the use of the Lovell peach reference for SNP discovery [13].

For clarity, the ‘Adafuel’ dataset was mapped only to the peach genome cv. Lovell from the National Center for Biotechnology Information (NCBI) and Joint Genome Institute (JGI) (Fig. 2C). Interestingly, both aligners exhibited comparable accuracy, yielding similar mapping results.

GMAP also excelled in transcript mapping (Fig. 2C). It successfully aligned a substantially higher number of transcripts from the peach reference transcriptome (RefTrans_V1; 23 390) compared to BLASTN (20 482 vs. 4902).

Altogether, GMAP consistently surpassed BLASTN across all tested scenarios, achieving significantly higher mapping rates and faster processing times (seconds vs. minutes) compared to BLASTN. Unmapped queries can be attributed to the stringent sequence identity and query coverage thresholds, set by default at 98% and 95%, respectively. Hits below these cutoffs are deemed unreliable and are discarded from the datasets.

To measure mapping accuracy, we aligned the nucleotide probes of the peach 18 K SNP array to the Pp_Lovell_NCBI_V2 reference and checked how often the genomic regions returned by GMAP and BLASTN contained the cognate SNP position. Among 16 095 probes aligned by both algorithms, 16 093 contained the correct SNP location. Only two discrepancies were observed, due to probes mapping to multiple genomic regions with similar sequence identity. The same experiment was carried out with almond 60 K and cherry 6 K SNP arrays, finding that all mapped SNPs (71 835 and 5492) were accurately positioned within the aligned intervals identified by both GMAP and BLASTN.

### Benchmarking the accuracy of PrunusMap

**Figure 3:**
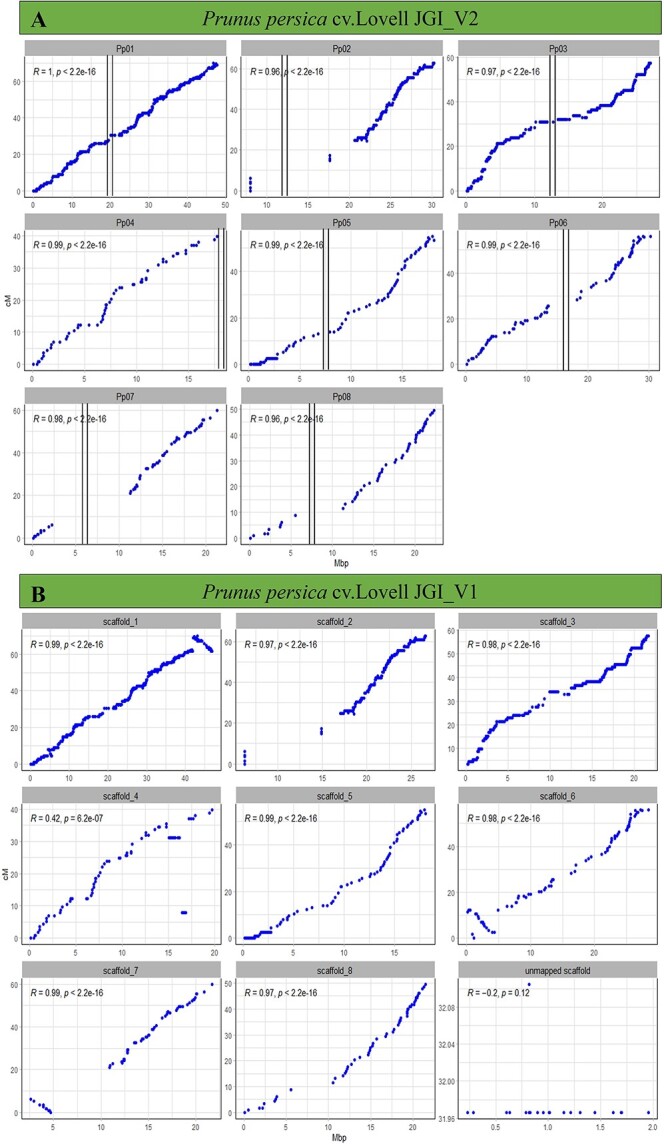
Relationship between the genetic and physical position of ‘Adafuel’ SNP markers within each pseudomolecule (chromosomes). For *P. persica* Pp_Lovell_JGI_V2 (A), pseudomolecules are referred to as Pp01 to Pp08, while in *P. persica* Pp_Lovell_JGI_V1 (B), they are labeled as scaffold_1 to scaffold_8. SNP markers were plotted according to their physical position on the peach genome reference (*x*-axis), and their genetic position was retrieved from the ‘Adafuel’ linkage map (*y*-axis). Vertical bars indicate the putative position of the centromeres, and R values correspond to the ‘Pearson’ correlation.

‘Adafuel’ marker sequences were aligned against the different databases of *P. persica* cv. Lovell using ‘bmap_align’. The resulting physical positions (in megabase pairs) were plotted against their genetic positions (in centimorgans), as reported by [14]. Notably, high collinearity was evident across all chromosomes, with ‘Pearson’ correlation coefficients ≥0.96 for reference Pp_Lovell_JGI_V2 (Fig. 3A). Overlapping gaps between genetic and physical positions were mainly observed in chromosomes Pp02, Pp07, and Pp08. These are explained by the uneven distribution of markers mapped in the Adafuel × Flordaguard population [14]. Note that there are no markers mapping on the short arm of Pp04. Interestingly, when Pp_Lovell_JGI_V1 was used as reference, inversions in scaffolds 1, 6, and 7 were revealed, as confirmed by Verde et al. [15] (Fig. 3B). Similarly, a misplaced contig appears near the centromere of scaffold 4. According to our results, these assembly conformations were corrected in V2, and 60 chromosomal SNPs previously located on unplaced scaffolds were also rectified. Comparable results were obtained for NCBI references, plotted in Figs S2 and S3.

### Highlighting protein alignment as a new key feature

**Figure 4:**
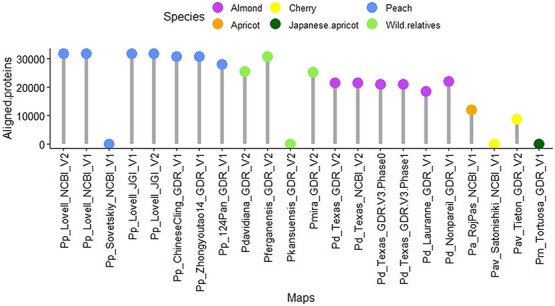
Cross-species protein mapping in PrunusMap. Protein sequences from peach cultivar Lovell (NCBI_V2) were mapped against reference genomes (maps) (*x*-axis) within PrunusMap. The *y*-axis represents the number of protein sequences with high-fidelity alignments.

PrunusMap extends BarleyMap by introducing a novel feature for protein sequence alignment. Indeed, the novel ‘Align_proteins’ tool drives protein-to-genome alignments. To benchmark ‘Align_proteins’, stringent criteria (identity ≥98% and coverage ≥95%) were employed to align complete protein sequences along with short peptides of 50 and 100 bp, against their respective reference genomes. Remarkably, this tool demonstrated high reliability, successfully aligning over 85% of protein queries (Fig. S4). We further assessed cross-species protein mapping. Here, we attempted to map protein sequences from a single species (e.g. peach cv. Lovell_NCBI_V2) against all reference genomes within PrunusMap (Fig. 4). This approach allows researchers to leverage well-annotated proteins for functional insights in species with limited annotations.

### Examining the insights provided by PrunusMap

**Figure 5:**
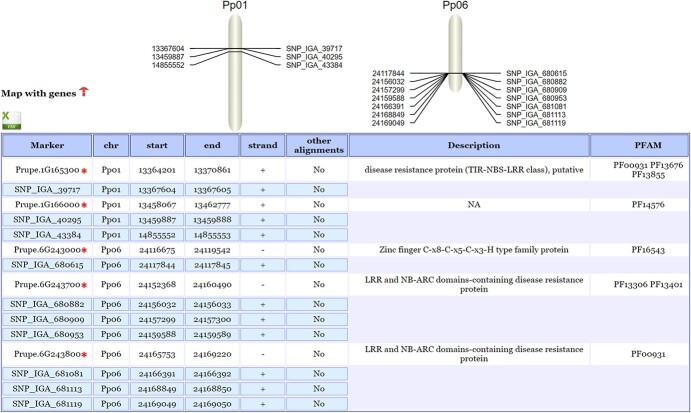
Illustration of PrunusMap functionalities. Genes marked with an asterisk correspond to nearby genes identified through the ‘Find marker’ tool.

The ‘Find markers’ tool successfully mapped 10 out of 11 user-provided markers linked to peach bacterial spot resistance to Pp_Lovell_JGI_V2 (Fig. 5**)**. The unmapped marker (SNP_IGA_46754) failed to meet minimum alignment quality thresholds. Mapped markers reside on chromosomes Pp01 and Pp06, flanking genes annotated with disease resistance protein domains (LRR and NB-ARC) (*Prupe.1G165300*, *Prupe.6G243700*, and *Prupe.6G243800*). These genes are known as major disease-resistance genes in plants and have been reported to be involved in pathogen recognition and innate immune responses in peach [16].

**Figure 6:**
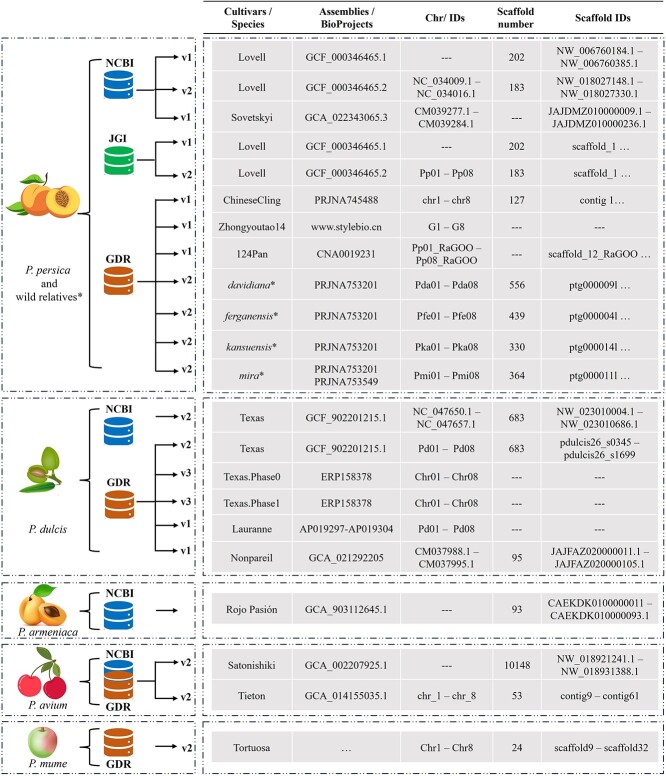
Illustration of PrunusMap databases (maps according to PrunusMap terminology). Please note that when ‘. . .’ is used, it means that IDs are not in consecutive order. However, when a range is used; for instance, NC_034009.1 – NC_034016.1 means the IDs are named in continued order. Abbreviations are as follows: Pp—*P. persica* (peach), Pd—*P. dulcis* (almond), Pa—*P. armeniaca* (apricot), Pav—*P. avium* (sweet cherry), and Pm—*P. mume* (Japanese apricot)*.* The asterisk (*) refers to closely related wild peach species.

## Discussion

In the following sections, we compare PrunusMap and other tools offering similar functionalities.

### Genome Database for Rosaceae and PrunusMap

The GDR serves as a central hub of the Rosaceae family. As a public repository, it provides access to multiple versions of genome assemblies enriched with gene descriptions, InterPro domains, and Gene Ontology (GO) and Kyoto Encyclopedia of Genes and Genomes (KEGG) pathway terms [10]. Moreover, it hosts collections of expressed sequence tags, full-length transcripts, metabolic pathways, maps, and quantitative and Mendelian trait loci linked to agronomically significant traits [10]. Beyond its role as a repository, the GDR provides analytical tools to explore genetic and genomic data (search, sequence retrieval, BLAST, synteny viewer, map viewer, Breeding Information Management System (BIMS).

While sharing similar purposes, PrunusMap is specifically designed to facilitate the identification of *Prunus* markers on both physical and genetic maps, providing a streamlined analysis that includes a detailed list of nearby genes and proteins. Although this functionality may seem to be fulfilled by GDR, there are key differences incorporated in PrunusMap.

First, regarding sequence homology searches, the GDR relies exclusively on BLAST, whereas PrunusMap employs the GMAP as its default aligner complemented with BLASTN. Based on our findings, GMAP consistently outperformed BLASTN in locating marker sequences (Figs 2 and S1). This performance gain is further accentuated when mapping transcripts, which require intron-aware alignments. These results agree with those reported in barley [11].

Second, when searching for molecular markers, the GDR supports an extensive list of parameters such as marker type, name, array, organism, chromosome, map, trait, and citation. PrunusMap further refined its search capabilities to align with our vision, offering a graphical visualization of marker locations on their corresponding chromosomes. This view is accompanied by a table summarizing the start and end positions of the markers, the strand, and, crucially, a list of annotated genes and proteins residing nearby. The strand orientation, absent in GDR, is particularly valuable for breeders as it can assist in designing Polymerase Chain Reaction (PCR) primers or Kompetitive allele specific PCR (KASP) markers, predicting missense mutations, and defining haplotypes. Finally, by displaying nearby genes on the same results table, PrunusMap eliminates the need to conduct multiple searches and navigate from one webpage to another. This streamlined approach enhances efficiency and simplifies the process for researchers and breeders alike.

Third, PrunusMap offers a significant advantage by enabling cross-species and intraspecies marker mapping across diverse genome versions and cultivars. This capability addresses the gene nomenclature discrepancy that already exists between different databases, such as Pp_Lovell_NCBI and Pp_Lovell_JGI. Additionally, many published studies often tend to focus on a single cultivar, resulting in other cultivars being overlooked. For instance, a marker closely linked to a desirable trait in one cultivar may exhibit a different position or allele in another cultivar. PrunusMap can handle this imbalance enabling breeders to explore several references in the same search. This, in turn, facilitates the identification of new sources of desirable traits. For example, the ability to map the 60 K Axiom markers on three different almond genome references (Texas, Lauranne, and Nonpareil) would enable breeders to easily explore the genetic diversity and relationships among these three cultivars.

Fourth, PrunusMap streamlines retrieving the physical and genetic positions of all markers from a given genetic map in a single operation, as shown here with ‘Adafuel’ markers, which support the analysis of genetic maps in a single step.

Finally, PrunusMap further enhances user experience by enabling the option to send search results via email. This feature is particularly helpful for large-scale analyses that may require extended processing times.

Overall, both the GDR and PrunusMap serve as valuable tools, each offering distinct, yet complementary features tailored to different user preferences.

### PeachVar-DB and PrunusMap

PeachVar-DB is a valuable resource for exploring the genetic makeup of a collection comprising 121 peach accessions and 21 wild relatives from the *Amygdalus* subgenus derived from the resequencing [7]. Users can get a broad overview by selecting a specific accession, delving into a specific genome region, or conducting a targeted gene-level analysis by providing the gene ID and features such as 5’ Untranslated Region (UTR), 3’UTR, Coding Sequence (CDS), or primary transcript. However, unlike PrunusMap, when selecting an accession or chromosome region, PeachVar-DB does not display information on nearby genes alongside the genetic variants. As with GDR, PeachVar-DB utilizes BLASTN for sequence similarity comparison but lacks graphical visualization of the mapping results. Additionally, PrunusMap introduced the ‘Align_proteins’ feature to perform protein-to-nucleotide alignments. This functionality complements nucleotide-based sequence searches, as it can map protein sequences annotated in species, which are often more conserved than gene sequences. Furthermore, in contrast to PrunusMap, PeachVar-DB does not support multiquery searches and file upload functionalities. Finally, it exclusively presents information on markers aligned to the peach reference genome v2.0.

### Conclusions and future directions

PrunusMap was developed to empower *Prunus* researchers with user-friendly analysis tools to support decision-making and accelerate breeding goals. We anticipate it will serve as a valuable tool for breeders in combination with GDR and PeachVar-DB. Furthermore, we expect PrunusMap to be continuously updated and expanded to cover other *Prunus* species upon demand from users. We welcome feedback and suggestions at compbio@eead.csic.es.

## Material and methods

### PrunusMap Web interface

PrunusMap is a freely accessible application created as a fork of Barleymap [11]. Its back-end functionality and interactivity are implemented in Python 2.6, relying on CherryPy to handle the user requests [17]. The front-end interface uses a Perl graphical library for chromosome visualization and intuitive interaction (https://github.com/pseudogene/genetic-mapper) (Fig. 1).

### Biological resources

PrunusMap stores and categorizes data into four different classes: databases, maps, datasets, and annotations.

#### Databases

Databases are FASTA-formatted genome sequence files that support sequence alignments. These genome references were sourced from NCBI [18], Phytozome (JGI) [19], and GDR [8–10] and indexed using ‘gmap_build’ and ‘makeblastdb’ from GMAP and BLASTN tools [20, 21]. Additionally, for protein mapping, they were indexed using MINIPROT v13.0 [22]. An example of database configuration is provided in Fig. S5.

Currently, PrunusMap hosts a comprehensive collection of 22 genomes of *Prunus* species all described in Fig. 6: several relevant *P. persica* cultivars (e.g. ChineseCling, Zhongyoutao14, 124Pan, and Sovetskiy); wild relatives such as *P. davidiana*, *P. ferganensis*, *P. kansuensis*, and *P. mira*; and other economically valuable species like almond (*P. dulcis*), sweet cherry (*P. avium*), apricot (*P. armeniaca*), and Japanese apricot (*P. mume*).

**Table 1:** Gene models (A) markers (B) and mapped protein sequences (C) housed in PrunusMap.

Species	Maps	A. Gene models		
		Total	IDs	References
Prunus persica and wild relatives^*^	Pp_Lovell_NCBI_V1	28 087	*PRUPE_ppa019766mg*	[15, 23]
	Pp_Lovell_NCBI_V2	25 030	*LOC18793189*	
	Pp_Lovell_JGI_V1	27 864	*ppa000003m.g*	
	Pp_Lovell_JGI_V2	26 873	*Prupe.1G000100*	
	Pp_ChineseCling_GDR_V1	26 335	*evm.TU.contig279.5*	[24]
	Pp_Zhongyoutao14_GDR_V1	30 181	*Pp01G000510*	[25]
	Pp_124Pan_GDR_V1	25 155	*P124PAN00019*	[26]
	Pp_Sovetskiy_NCBI_V1	128	*gene-ndhA*	[27]
	Pdavidiana_GDR_V2^*^	27 236	*Pda01g0001*	[28]
	Pferganensis_GDR_V2^*^	28 587	*Pfe01g0001*	
	Pkansuensis_GDR_V2^*^	26 986	*Pka01g0001*	
	Pmira_GDR_V2^*^	28 519	*Pmi01g0001*	
***Prunus dulcis***	Pd_Lauranne_GDR_V1	23 266	*Prudu_020920_v1.0*	[29]
	Pd_Nonpareil_GDR_V1	45 581	*L3X38_000408*	[30]
	Pd_Texas_NCBI_V2	25 445	*LOC117629531*	[31]
	Pd_Texas_GDR_V2	27 042	*Prudul26A002130*	
	Pd_Texas_GDR_V3.Phase0	29 145	*TexasF0_G5*	[32]
	Pd_Texas_GDR_V3.Phase1	30 150	*TexasF1_G30142*	
***Prunus armeniaca***	Pa_RojPas_NCBI_V1	52 344	*Gene-CURHAP_LOCUS15*	[33]
***Prunus avium***	Pav_Satonishiki_NCBI_V1	28 800	*LOC110751831*	[34]
	Pav_Tieton_GDR_V1	39 984	*FUN_000009*	[35]
***Prunus mume***	Pm_Tortuosa_GDR_V1	29 706	*PmuVar_Chr2_0001*	[36]
SNP Arrays	Total	B. Markers		
		IDs		References
IRSC 9 K peach	9000	SNP_IGA_134631		[37]
		snp_scaffold_1_46157131		
		Pp8Cl		
		RosCOS1338-411		
IRSC 18 K peach	18 000	SNP_IGA_679		https://www.rosaceae.org/organism/26133
		Peach_AO_0000136		
		snp_scaffold_8_17395002		
		RosCOS1549-533		
IRSC 6 K cherry	5696	RosCOS1139-146_snp_sweet_cherry_Pp1_43832684		[13]
Axiom 60 K	71 846	AX-158803044		[12]
Adafuel	7831	Pp01_10008318_YC		[14]
Maps	Total	C. Proteins UniProt		
		IDs		References
Pp_Lovell_NCBI_V2	38 303	E3W0H3_PRUPE		[15]
Pdavidiana_GDR_V2	229	A0A385H4F7_9ROSA		[28]
Pferganensis_GDR_V2	87	A0A6B9IN88_9ROSA		
Pd_Lauranne_GDR_V1	39 030	A0A1W6CB65_PRUDU		[29]
Pd_Nonpareil_GDR_V1	38 377			[30]
Pd_Texas_NCBI_V2	42 636			[31]
Pd_Texas_GDR_V3.Phase0	39 243			[32]
Pd_Texas_GDR_V3.Phase1	39 222			
Pa_RojPas_NCBI_V1	43 609	A0A6J5TF90_PRUAR		[33]
Pav_Satonishiki_NCBI_V1	29 660	A0A6P5SIM1_PRUAV		[34]
Pav_Tieton_GDR_V1	27 102			[35]
Pm_Tortuosa_GDR_V1	225	A0A2H4N340_PRUMU		[36]

The asterisk ^*^ refers to closely related wild peach species belonging to the genus *Prunus* in the Rosaceae family.

#### Maps

Maps are files designed to store the positional arrangement, whether physical or genetic, of sequences derived from databases. PrunusMap employs standardized map identifiers, combining species abbreviations, cultivar names (if applicable), genome source, and version information. This format facilitates efficient map identification (e.g. Pp_ChinesCling_gdr1 represents *P. persica* cultivar ‘ChineseCling’, genome v1 downloaded from GDR. A detailed list of maps is available in the help section https://prunusmap.eead.csic.es/prunusmap/help.

#### Standalone version

Secondary tools such as ‘bmap_align_to_db’ and ‘bmap_align_to_map’ are only available in the standalone version, which can be installed following the instructions in the GitHub repository.

#### Datasets

PrunusMap datasets are genes, molecular markers, and UniProt proteins, often associated with AlphaFold structural models [21]. Each dataset is a collection of one of these classes along with their precomputed map positions, determined through sequence alignment against the reference database. Genes and markers were aligned using GMAP and/or BLASTN, while proteins were mapped with MINIPROT [22]. See Table 1 for a summary of gene models, markers, and lifted-over proteins.

PrunusMap provides a comprehensive collection of SNP markers, including the International Rosaceae SNP Consortium (IRSC) peach 9 K and 18 K arrays, the IRSC 6 K array for sweet cherry, and the Axiom 60 K Chip for almond. These datasets were sourced from the GDR (https://www.rosaceae.org/organism/26133) with the commitment to expand the collection based on user requests. The peach 9 K array originated from resequencing 56 peach accessions, with the 18 K chip built upon it. The cherry 6 K array was developed based on a detection panel of 16 sweet and eight sour cherry accessions, while the Axiom 60 K was derived from whole-genome resequencing of 81 almond genomes [12]. Notably, all these markers have been mapped across all reference genomes within PrunusMap, empowering users to efficiently identify the precise location of any marker on any available reference genome.

Genetic markers for the hybrid peach-almond rootstock ‘Adafuel’ were retrieved from the maps resulting from the analysis of the Adafuel × Flordaguard population [14]. Only SNPs from the ‘Adafuel’ linkage map were selected as they covered the eight-linkage group. Conversely, only four groups were constructed for ‘Flordaguard’. Currently, this is the only dataset in PrunusMap featuring genetic positions.

#### Annotations

Gene datasets were enriched with functional annotations from InterPro and Pfam databases [38, 39]. To ensure the highest accuracy, annotations were limited to references *P. persica* (Pp_Lovell_JGI_V2) and *P. dulcis* (Pd_Texas_GDR_V2).

#### PrunusMap commands: navigating the toolkit

PrunusMap offers a variety of Web and standalone tools, which are summarized below. While the former are publicly accessible, standalone tools require local installation and configuration of PrunusMap. Check the repository https://github.com/eead-csic-compbio/prunusmap_web and the help section at https://prunusmap.eead.csic.es/prunusmap/help for more details (Fig. 1).

#### Web-based tools

Markers can be searched using different inputs: FASTA-formatted genomic and protein sequences, IDs, or positions. This is facilitated by the following standalone tools, which can examine up to 10 000 entries on single or multiple maps.

- ‘bmap_align’: aligns FASTA sequences to reference databases using GMAP, BLASTN, or both [18, 21]. Initially, queries are searched using GMAP with BLASTN taking over if no matches are found. This iteration continues until either all queries are aligned, or no additional databases are available. The default parameters are set as minimum identity = 98% and minimum coverage = 95% but can be customized.

- ‘bmap_align_prot’: Similar to bmap_align, this tool aligns amino acid sequences considering splicing and frameshifts. The adjustable identity and query coverage cutoffs were set as 98% and 95% by default to ensure accurate protein hits.

- ‘bmap_find’: takes a list of query IDs and retrieves their alignment positions from the pre-computed datasets listed in Table 1.

- ‘bmap_locate’: locates features (genes, markers, and/or proteins) based on their chromosomal or scaffold positions. The input requires a list formatted with chromosome/scaffold names and base-pair/centimorgan positions in-line (e.g. Chr2 12 496 912).

For each tool, users can optionally retrieve lists of nearby genes and/or markers within a customizable radius (in base pairs) based on their specific needs.

#### PrunusMap output

The output is provided through the Web interface or conveniently sent to an email address for ease of interpretation and storage. Within the Web interface, the results are displayed in two formats: as a graphical representation of the genome, highlighting the query locations and as a downloadable Comma Separated Values (CSV) file (Fig. 1). An additional table showcasing the position of nearby markers, genes, or proteins is also provided. The graphical interface that depicts chromosomes as vertical bars offers functionality comparable to classic genome browsers, allowing users to visualize genomic features relative to their chromosomal positions (Fig. S6). The search radius for neighboring features can be tailored and defined either in base pairs (bp) or in centimorgans (cM), depending on the underlying selected map (Fig. S7). Additional details are provided in the help section: https://prunusmap.eead.csic.es/prunusmap/help.

#### Showcase analysis

To benchmark PrunusMap, we analyzed relevant markers documented in the literature. For instance, [40] reported two quantitative trait loci (QTLs) linked to fruit resistance to bacterial spot infection (‘Xap.PpOC-1.2’ and ‘Xap.PpOC-1.6’), sitting on chromosomes 1 and 6. Eleven SNPs in close proximity to these QTLs were used to design KASP markers, resulting in a 44% reduction in seedling planting [40]. In this context, we tested the ‘Find markers’ Web tool in order to locate the 11 SNPs that correspond to the following 9 K peach array markers: SNP_IGA_39717, SNP_IGA_40295, SNP_IGA_43384, SNP_IGA_46754, SNP_IGA_680615, SNP_IGA_680882, SNP_IGA_680909, SNP_IGA_680953, SNP_IGA_681081, SNP_IGA_681113, and SNP_IGA_681119.

## Supplementary Material

Web_Material_uhae301

## Data Availability

PrunusMap is freely accessible at https://prunusmap.eead.csic.es. Instructions for configuring and using the Web application can be found at https://github.com/eead-csic-compbio/prunusmap_web. All sets of curated SNP markers mapped using PrunusMap are accessible at https://github.com/eead-csic-compbio/prunusmap_web/tree/master/download.
