# Supplementary material for: Mapping the genomic landscape of Prunus spp. with PrunusMap
Source: Hortic Res. 2024 Oct 24;12(2):uhae301. doi: 10.1093/hr/uhae301 (PMC11822410; doi:10.1093/hr/uhae301)
Supplement: Web_Material_uhae301 [file web_material_uhae301.pdf]

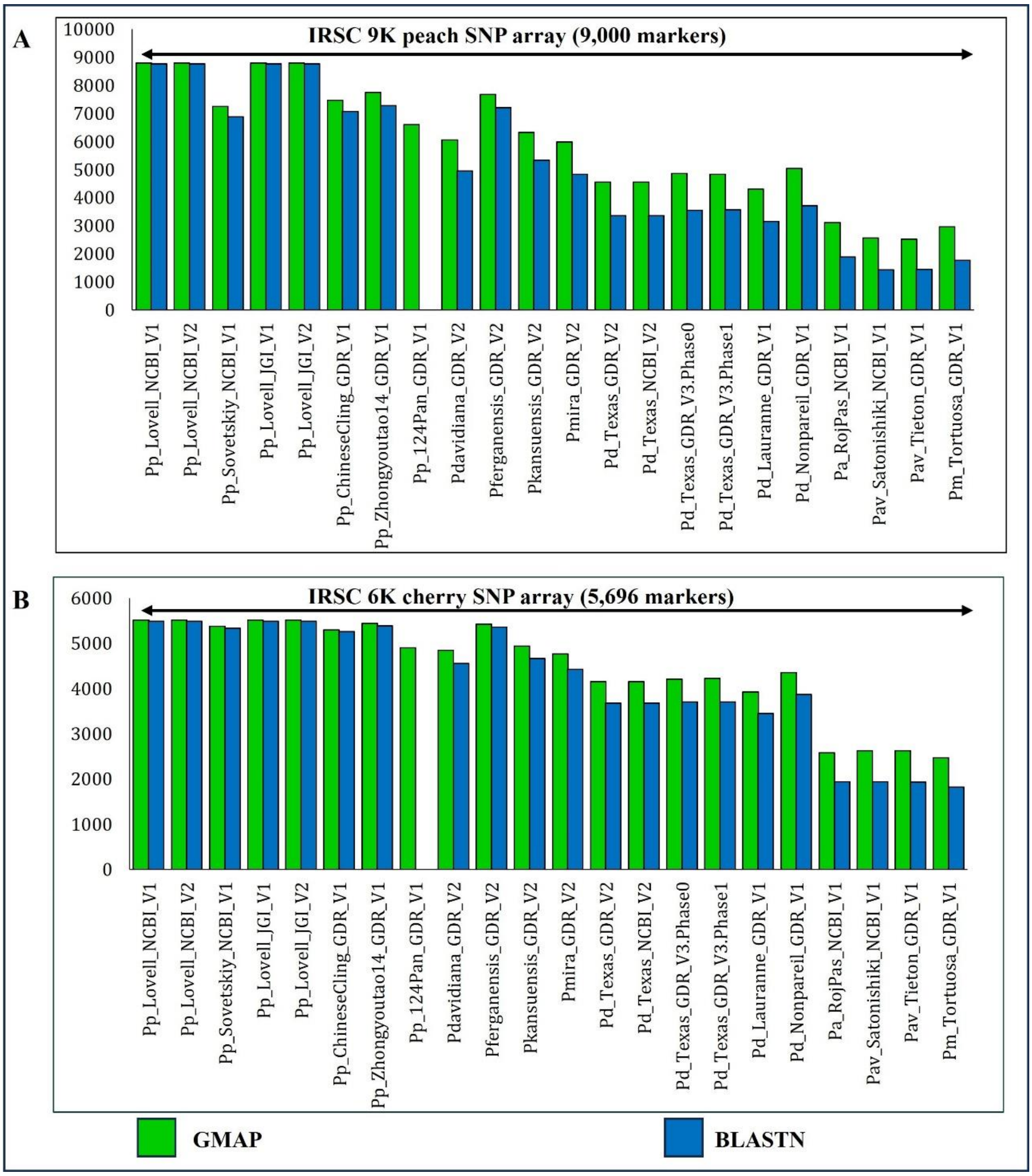

Supplemental Figure S1. Performance comparison of GMAP and BLASTN aligners. (**A**) and (**B**) correspond to the alignment of the IRSC 9K peach SNP and IRSC 6K cherry arrays against all PrunusMap databases, respectively. The x-axis refers to the different databases (maps according to PrunusMap terminology) used for the alignment while the y-axis corresponds to the number of sequences. Green and blue bars correspond to the aligned hits using GMAP and BLASTN, respectively.

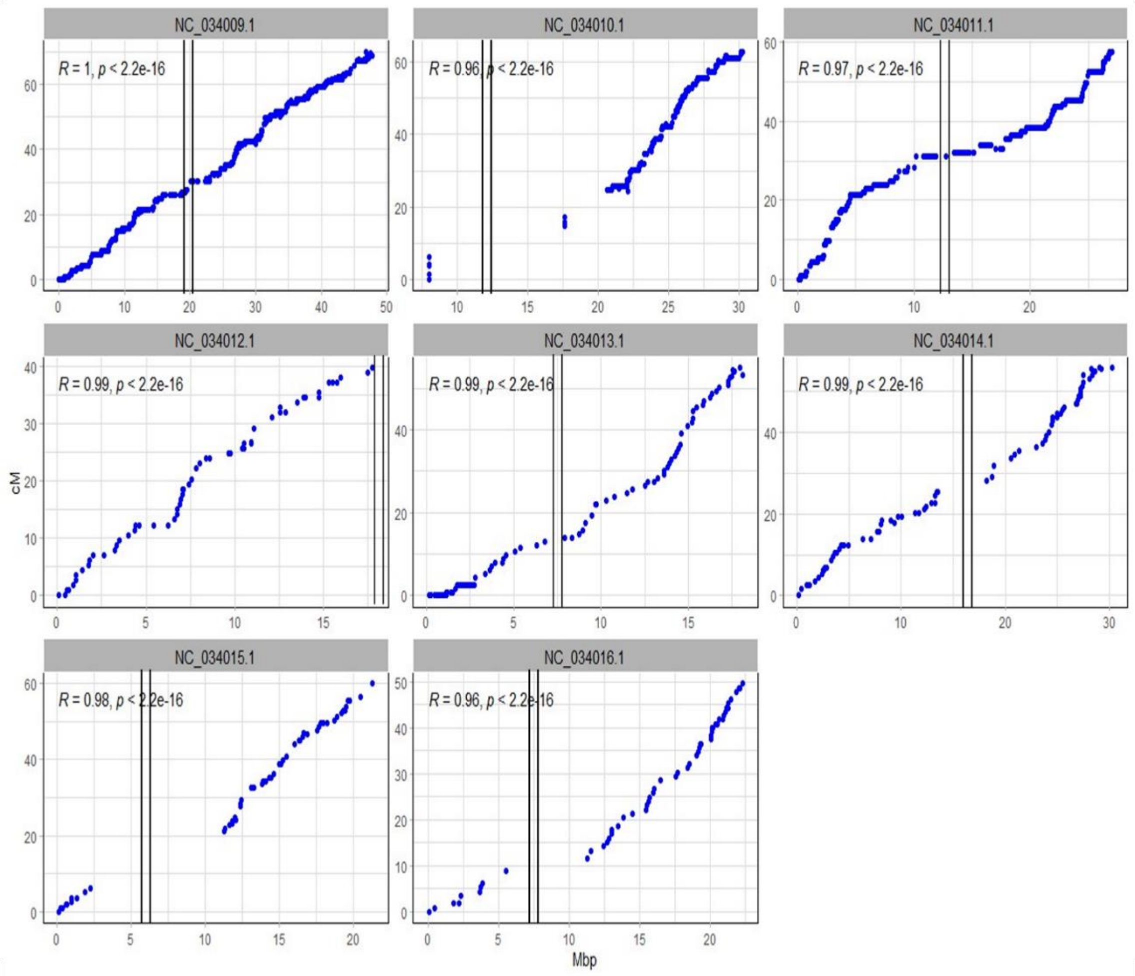

Supplemental Figure S2. Relationship between genetic and physical position of 'Adafuel' SNP markers mapped to peach reference genome NCBI_V2. Pseudomolecules are referred to as NC_034009.1 to NC_034016.1. Markers were plotted according to their physical position in Mbp on peach NCBI_V2 (x-axis) and their genetic position in cM (y-axis). Vertical bars indicate putative position of the centromeres.

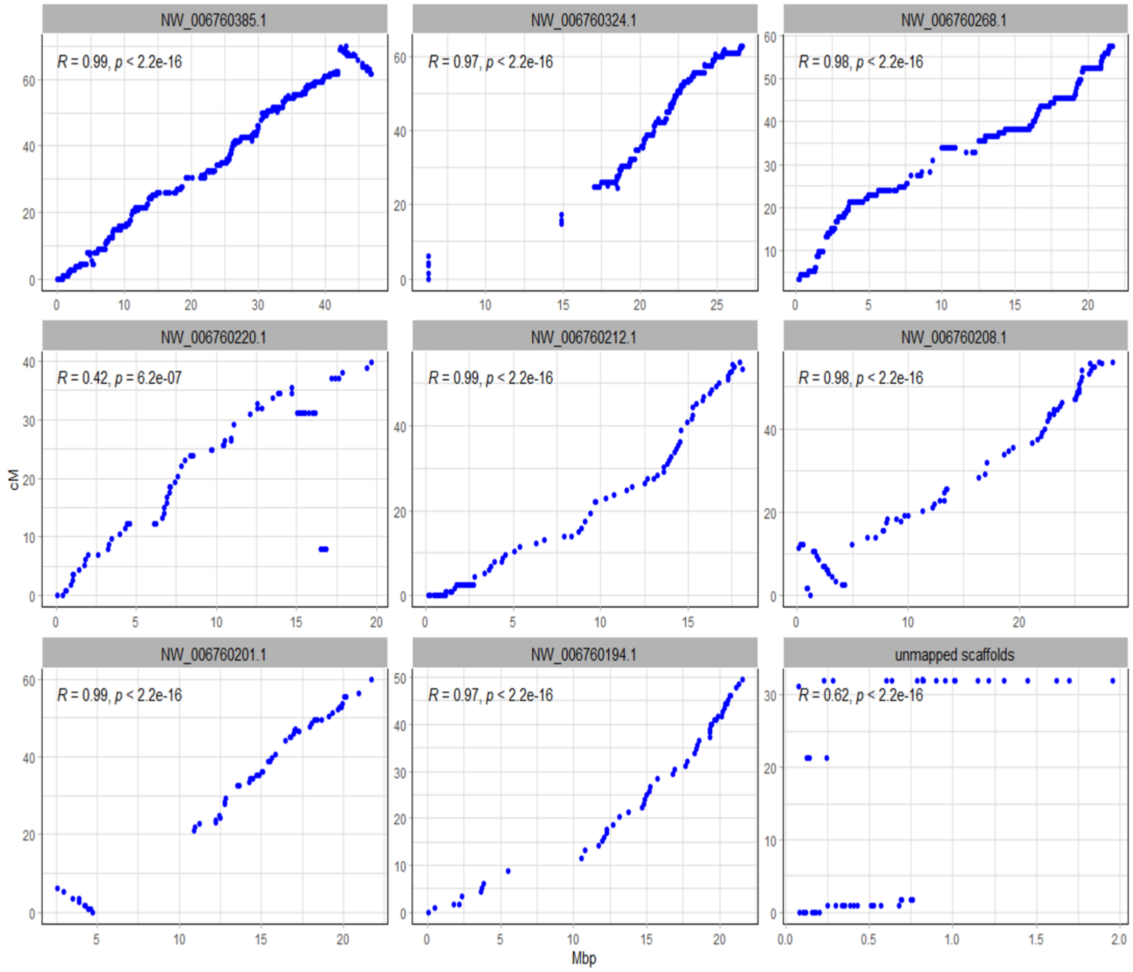

Supplemental Figure S3. Relationship between genetic and physical position of 'Adafuel' SNP markers mapped to peach reference genome NCBI_V1. Scaffolds are referred to as NW_006760385.1 and so forth. Markers were plotted according to their physical position in Mbp on peach NCBI_V1 (x-axis) and their genetic position in cM (y-axis) from the "Adafuel" genetic map.

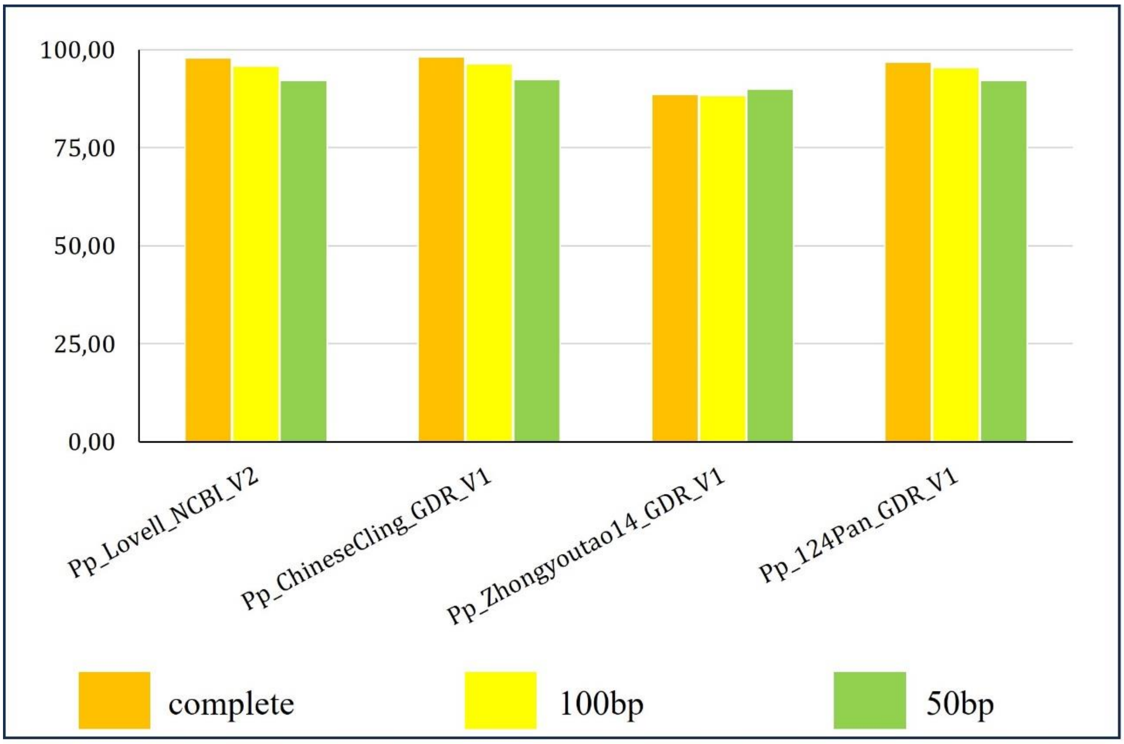

Supplemental Figure S4. Relationship to align complete protein sequences along with short peptides of 50 and 100 bp, against their respective reference genomes. Remarkably, this tool demonstrated high reliability, successfully aligning over 85% of protein queries.

A

> Databases configuration

# name	unique_id	type
Pp_Lovell_NCBI_V2	Prunus_persica_NCBIv2	std
Pd_Texas_GDR_V2	Prunus_dulcis_Texas_GDRv2	std

B

> Maps configuration

#name	id	has_cm	has_bp	default_pos_type	map_type	search_type	db_list	folder_name	main_datasets
Pp_Lovell_NCBI_V2	pp_ncbi2	cm_true	bp_true	bp	physical	greedy	Prunus_persica_NCBIv2	pp_ncbi2	ncbi2_lovell_genes
Pd_Lauranne_GDR_V1	pd_Texas_gdr1	cm_true	bp_true	bp	physical	greedy	Prunus_dulcis_Texas_GDRv2	pd_lauranne	lauranne_genes

C

> Datasets configuration

> Gene models

#name	unique_id	type	filename	file_type	db_list	synonyms_file	records_prefix
ncbi2_lovell_genes	ncbi2_genes	gene	~/Prunus_persica_NCBIv2. genomic.gff.gz	gtf	Prunus_persica_NCBIv2	no	no

> Genetic markers

9K_peach	9K_peach	genetic marker	~/IRSC_9k_peach_SNP.array.fna	fna	Prunus_persica_NCBIv2, Prunus_persica_NCBIv1	no	no

D

> Map example

Pd01	1	43698516
Pd02	2	24875702
Pd03	3	23079971
Pd04	4	21880570
Pd05	5	17832838
Pd06	6	29142197

E

> Dataset example

Marker	chr	snp_start	snp_end	strand	genetic_pos	multiple_positions	other_alignment
SNP_IGA_679	Pp01	94644	94645	+	No	No	No
Peach_AO_0000136	Pp01	97464	97465	+	No	No	No
Pp3C1	Pp01	35185953	35185954	-	No	No	No
RosCOS1462-378	Pp06	3254055	3254056	-	No	No	No

Supplemental Figure S5. An overview of biological resource configuration in the PrunusMap framework. Panels **A**, **B** and **C** represent the file structure of databases, maps and datasets respectively. Panels **D** and **E** showcase examples of maps and datasets.

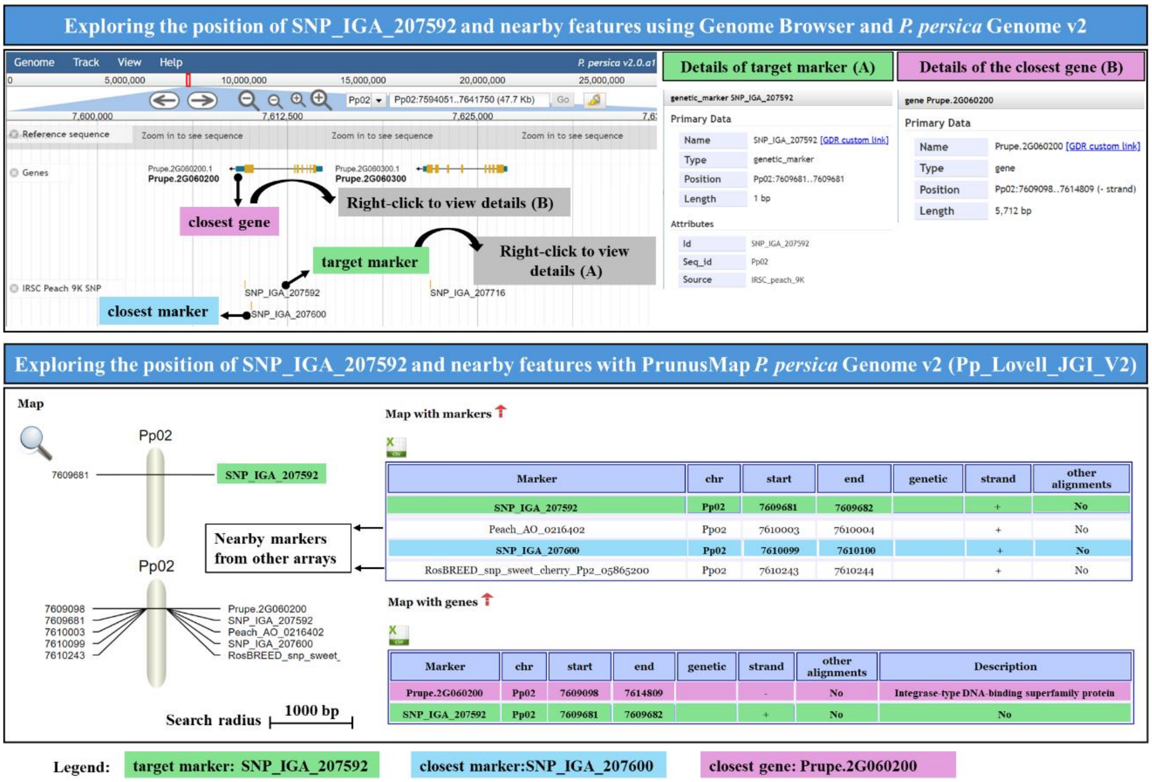

Supplemental Figure S6. Side-by-side view of Genome Browser and PrunusMap using *Prunus persica* genome, cv.Lovell v2. The target maker "SNP_IGA_207592" from the peach 9K array is highlighted in green, while the closest marker and gene to the target are highlighted in light blue and purple, respectively. In a classical genome browser, these features are displayed in a horizontal format, requiring a right-click to view their corresponding details (gray boxes). PrunusMap offers a streamlined approach by displaying on a vertical bar, accompanied by two separate tables: one for nearby markers and another for genes with their corresponding annotation. The marker table includes not only markers from the same array as the query, but also nearby markers from other arrays available in PrunusMap. The search radius in PrunusMap is equivalent to zooming in or out in a browser allowing users to adjust the search range.

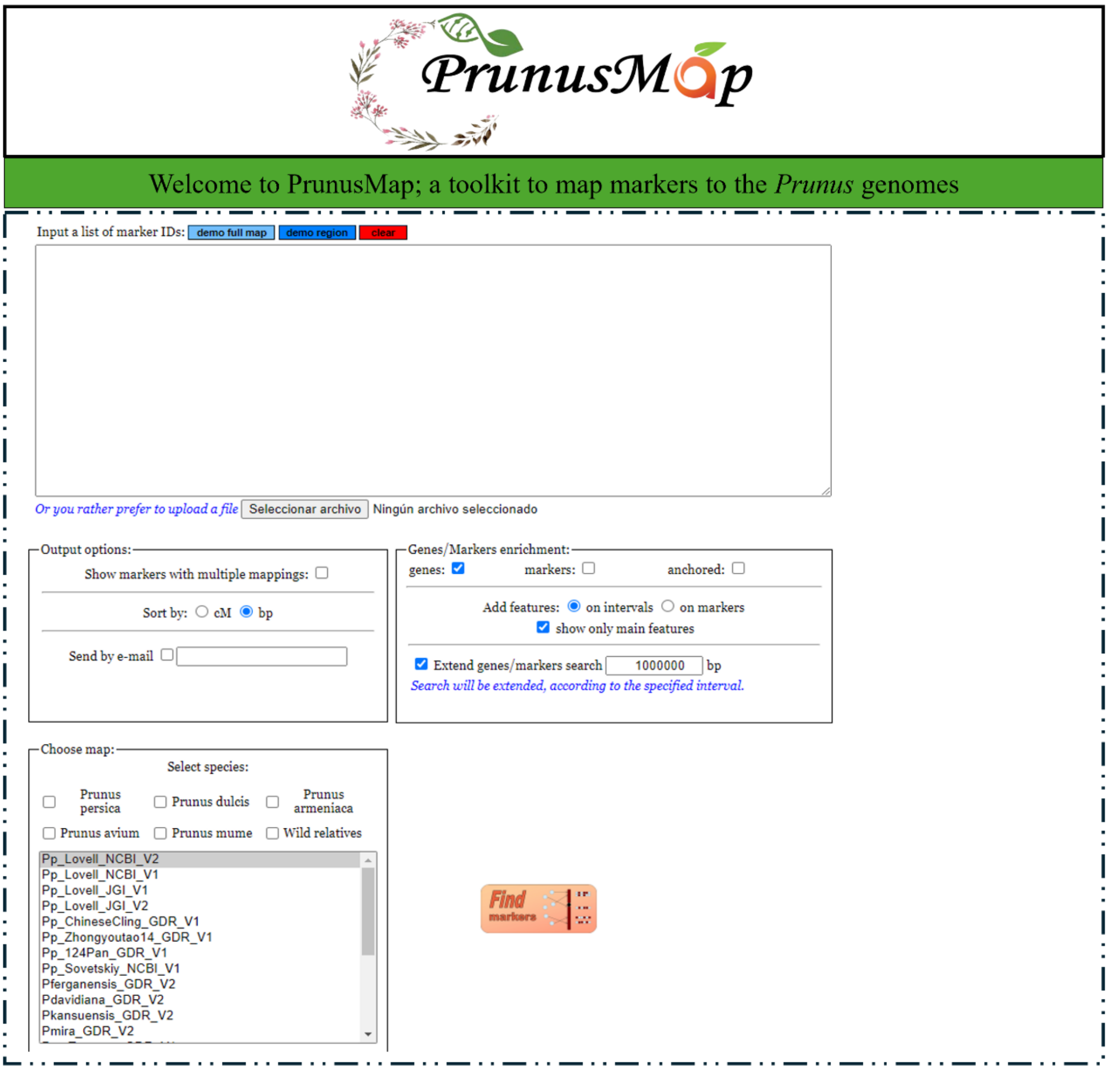

Supplemental Figure S7. Screen capture of PrunusMap Web portal. Queries can be entered directly in the input box or uploaded as a file. The output options box gives the users the option to provide their email and to sort the results by their physical (bp) or genetic positions (cM). The Genes/Marker enrichment panel allows users to retrieve the adjacent features (genes, markers and/or proteins) within a user-customized interval.
